# Novel Bayes Factors That Capture Expert Uncertainty in Prior Density Specification in Genetic Association Studies

**DOI:** 10.1002/gepi.21891

**Published:** 2015-02-27

**Authors:** Amy V. Spencer, Angela Cox, Wei‐Yu Lin, Douglas F. Easton, Kyriaki Michailidou, Kevin Walters

**Affiliations:** ^1^ School of Mathematics and StatisticsUniversity of SheffieldSheffieldUK; ^2^ Department of OncologySheffield Cancer Research CentreUniversity of Sheffield Medical SchoolSheffieldUK; ^3^ Department of NeurosurgeryChang Gung Memorial HospitalTaoyuan CountyTaiwan; ^4^ Department of Public Health and Primary CareCentre for Cancer Genetic EpidemiologyUniversity of CambridgeCambridgeUK; ^5^ Department of OncologyCentre for Cancer Genetic EpidemiologyUniversity of CambridgeCambridgeUK

**Keywords:** expert knowledge, elicitation, sensitivity, hyperparameter, flexibility, single‐nucleotide polymorphism, filtering, empirical, fine mapping

## Abstract

Bayes factors (BFs) are becoming increasingly important tools in genetic association studies, partly because they provide a natural framework for including prior information. The Wakefield BF (WBF) approximation is easy to calculate and assumes a normal prior on the log odds ratio (logOR) with a mean of zero. However, the prior variance (*W*) must be specified. Because of the potentially high sensitivity of the WBF to the choice of *W*, we propose several new BF approximations with  logOR ∼N(0,W), but allow *W* to take a probability distribution rather than a fixed value. We provide several prior distributions for *W* which lead to BFs that can be calculated easily in freely available software packages. These priors allow a wide range of densities for *W* and provide considerable flexibility. We examine some properties of the priors and BFs and show how to determine the most appropriate prior based on elicited quantiles of the prior odds ratio (OR). We show by simulation that our novel BFs have superior true‐positive rates at low false‐positive rates compared to those from both *P*‐value and WBF analyses across a range of sample sizes and ORs. We give an example of utilizing our BFs to fine‐map the *CASP8* region using genotype data on approximately 46,000 breast cancer case and 43,000 healthy control samples from the Collaborative Oncological Gene‐environment Study (COGS) Consortium, and compare the single‐nucleotide polymorphism ranks to those obtained using WBFs and *P*‐values from univariate logistic regression.

## Introduction

Recently, several methods have been published for analyzing genotype data at the fine‐mapping level [Maller et al., 2012; Udler et al., 2009; Vignal et al., 2011]. These data consist of hundreds or thousands of single‐nucleotide polymorphisms (SNPs) in a small region of the genome, where associations with a disease have previously been found, commonly in a genome‐wide association study (GWAS). Bayesian methods [Stephens and Balding, 2009] have the advantage of naturally allowing for the inclusion of prior functional genetic information which could inform the probability of an SNP being causal. This could help to overcome the problems faced in fine‐mapping, where it is expected that the causal SNPs not yet identified are likely to have small effect sizes (odds ratios [ORs] of the order of 1.1 or less) and the results are likely to be confounded by high levels of short‐range linkage disequilibrium (LD). Furthermore, some causal SNPs may have low minor allele frequencies (MAFs). These factors make it difficult to identify causal SNPs, even using the tens of thousands of subjects that are currently being analyzed by international consortia.

We previously carried out a thorough investigation of several different frequentist fine‐mapping filtering methods [Spencer et al., 2014]. Filtering is a general framework in which the SNPs are ranked according to a given statistic, a threshold value of that statistic is chosen and SNPs with a value below this threshold are removed from the set of candidate causal SNPs. This results in a more manageable number of SNPs which can be investigated for causality in further biological tests, such as the analysis of gene expression in cell lines. The Bayes factor (BF) is a Bayesian statistic which can be used for filtering [Kass and Raftery, 1995], and it has already been used as a tool for use in genetic association analyses [Stephens and Balding, 2009; Wakefield, 2008, 2009]. The calculation of BFs is now implemented in genetic analysis software such as snptest2 [Marchini et al., 2007] and their use is becoming increasingly popular as a filter in fine‐mapping studies [Maller et al., 2012]. The BF is a ratio, which compares the probabilities of the data under two models or hypotheses. In this setting, they compare two models, one in which an SNP is not causally associated with a disease and the other in which it is. They can be used alone or used to update prior odds of the SNP being causal. BFs often have to be approximated due to intractable integrals and, although current methods of approximation are good, they restrict the form of the prior on the effect size.

We consider the use of Wakefield's approximate BF [Wakefield, 2008, 2009], which requires that each SNP has a prior on its log odds ratio (logOR) of the form N(0,W) for some fixed *W*. When eliciting a value of *W* from experts it may be the case that there is some uncertainty in *W*, perhaps because several quantiles have been elicited giving inconsistent values of *W*. Because of this we examine several families of prior distributions on *W*, which result in novel tractable BFs. Such priors on *W* can be thought of as providing a weighted average of the BF over each value in the support of *W*. We examine the properties of these priors on *W* and give the novel BF approximations in forms that are easily calculable in commonly used software. Although this removes the problem of specifying a fixed *W* value, most of the novel BFs require hyperparameters to be specified. We demonstrate how appropriate values may be obtained via expert elicitation.

The BFs we describe could be used in any genetic association study, but we give an example with simulated fine‐mapped data to show how effective the use of these BFs can be in filtering. We compare the results to those using the Wakefield BF (WBF) and examine the effect of the choice of hyperparameters. We give an example of eliciting the prior hyperparameters and using the BFs as a fine‐mapping tool using breast cancer case‐control data from an international consortium. We are able to show that our methods may be used to describe a variety of uncertainties and appropriately incorporate these into a BF analysis. Not only this, but they can potentially produce better results than if the uncertainty was not taken into account.

## Materials and Methods

### Bayes Factors and the Wakefield Approximation

BFs compare the probability of the observed data under two models or hypotheses. For our purposes, the BF can be defined as

(1) $$\mathrm{BF}=\frac{P(data|H_{1})}{P(data|H_{0})}.$$

BFs are also used to update prior odds (δ/(1−δ)) to posterior odds (Δ/(1−Δ)) via Δ/(1−Δ)=δ/(1−δ)× BF , where in our case Δ and δ are the posterior and prior probabilities of “true” association, respectively. By “true association,” we mean causally linked to disease risk rather than being associated through LD or sampling variation. Here, a BF greater than one indicates that the data are more likely under the alternative than the null hypothesis. BFs require the specification of a likelihood and prior on all model parameters. Both BFs and posterior probabilities can be used to fine‐map genomic regions in case‐control studies by using the likelihood from a logistic regression model. For SNP *i* in single‐SNP logistic regression models, the probability ($y_{ij}$) of subject *j*, with $x_{ij}$ copies of the minor allele, being a case is

(2) $$y_{ij}=\frac{e^{\beta_{0i}+\beta_{1i}x_{ij}}}{1+e^{\beta_{0i}+\beta_{1i}x_{ij}}}.$$

With this definition, $\beta_{1i}$ can be interpreted as the SNP‐specific per‐allele natural logarithm of the OR comparing the minor to the major allele. For SNP *i*, BF_*i*_ is calculated comparing the hypotheses $H_{0}:\beta_{1i}=0$ and $H_{1}:\beta_{1i}\neq 0$ [Stephens and Balding, 2009].

The BF, as given in Equation (1), is the ratio of marginal likelihoods which can lead to intractable integrals for many prior densities. For nontractable BFs, it is common to use a Laplace approximation [Kass and Raftery, 1995]. The Laplace approximation is implemented in software packages, including snptest2 [Marchini et al., 2007]. Wakefield [2008, 2009] derived a tractable approximation to the BF (which we abbreviate as WBF). We found excellent agreement between the WBF and Laplace approximations from snptest2 for sample sizes ⩾10, 000 for a variety of ORs and MAFs (data not shown). Both methods are based on asymptotic approximations and, given the large sample sizes in the types of dataset we consider, should provide accurate approximations to the true BF.

Using the definition of the BF in Equation (1), the Wakefield approximate BF is

(3) $$\mathrm{WBF}=\sqrt{\frac{V}{V+W}}\exp\left(\frac{{\hat{\beta_{1}}}^{2}W}{2V(V+W)}\right).$$

In Equation (3), β_1_, the logOR of causal SNPs in the genomic region under consideration, is assumed to follow a normal distribution given by $\beta_{1}∼N\left(0,W\right)$. $\hat{\beta_{1}}$ is the maximum likelihood estimator (MLE) of β_1_. Rather than consider the logistic likelihood, Wakefield used the asymptotic distribution of the MLE: $\hat{\beta_{1}}∼N\left(\beta_{1},V\right)$ which leads to the WBF given in Equation (3). Note that the WBF we specify in Equation (3), and use in the rest of this paper, is the reciprocal of the WBF given by Wakefield [2009].

### Motivation for the Study

To use the WBF, one needs to specify *W* (e.g., through elicitation) and be prepared to accept that the prior distribution of the logOR is Gaussian. For a percentile $\beta_{1,p}$, such that $p(\beta_{1}<\beta_{1,p}|\beta_{1}∼N\left(0,W\right))=p$, *W* is calculated using $W={\left\{\beta_{1,p}/\Phi^{-1}\left(p\right)\right\}}^{2}$, where Φ is the distribution function of the standard normal distribution (Wakefield, 2009). When performing elicitation about *W* with an expert, they may express some uncertainty about the value of *W*. For example, the expert may believe that the 80th percentile of the prior distribution for the OR is between 1.05 and 1.3 which implies that 0.003≤W≤0.1. As a result, we investigated how sensitive the WBFs are to the choice of *W*. We found the results to be highly dependent on the choice of *W*. We therefore wanted to allow for uncertainty about *W* in the BF calculations. We retained the normal density for the prior for β_1_ and considered three different parametric families of priors for *W* that yield BFs that should be flexible enough to capture expert uncertainty in *W*. We also considered an additional prior that may be useful in some scenarios. We wanted priors that led to BFs that could be easily calculated and this informed our choice of priors. We go on to show that these priors have desirable properties in the context of fine‐mapping and carry out sensitivity analysis to demonstrate the effect of the prior parameters. We give suggestions for how prior hyperparameters might be elicited and in the supplementary material we provide **R** code to calculate the new BFs.

### Novel BFs Allowing for Uncertainty in *W*

We have derived four forms for priors on *W* which are given (up to proportionality) in Table 1. Three of these forms, the power, hybrid, and reciprocal priors, use the genotype data through *V*. The dependence of the prior for *W* on *V* is purely for mathematical convenience to yield tractable integrals. Therefore, they are not true priors but we show that in practice the values of *V* likely to be encountered in large association studies have very little impact on the prior density of *W*. We also provide one prior on *W*, the exponential prior, which does not depend on the genotype data.

**Table 1:** Prior densities for *W*

Name of prior	f(W)∝	Restrictions on hyperparameters
Power	${\left(V+W\right)}^{k}$	$k<-\frac{1}{2}$
Exponential	$\exp\left(-cW/2\right)$	c>0
Hybrid	${\left(V+W\right)}^{k}\exp\left(-\frac{d}{2(V+W)}\right)$	$d>-\hat{\beta_{1}},k<-1$
Reciprocal	$\frac{1}{\left(V+W\right)}\exp\left(-\frac{\left(V+W\right)}{2}\right)$	

Proportional density functions for each of the four prior forms (applies for 0<a≤W≤b).

**Figure 1:**
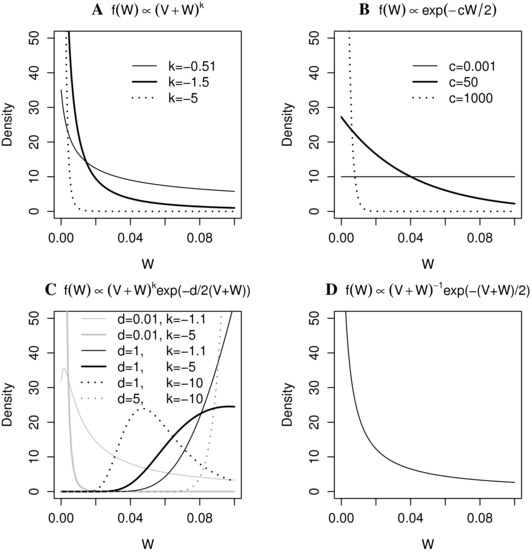
Densities of three families of tractable priors and one specific prior for f(W)(0<W≤0.1). β_1_ is logOR with $\beta_{1}∼N\left(0,W\right)$ and a value of V=0.003 is used in all plots.

Each of the power, exponential, and hybrid priors are really families of priors because they depend on hyperparameters, whereas the reciprocal prior takes a single density. These hyperparameters are represented by *k*, *c*, and *d*, and all priors have a support 0<a≤W≤b. We suggest choosing the values of *a*, *b*, *c*, *d*, and *k* via expert elicitation. Figure 1 shows the densities of some possible priors and hence the range of prior beliefs they can accommodate. We derive the approximate BFs relating to these priors in the supplementary material. All four new BFs can be easily calculated in **R** [R Core Team, 2012] (code is provided in the supplementary material). It should be noted that what we term the hydrid prior is identical to a shifted inverse gamma distribution on a restricted support.

### Eliciting Hyperparameters of the Priors for *W*

Our new BFs avoid the problem of specifying a fixed value for *W*, but instead require other hyperparameter specification for most of the BFs suggested. The hyperparameters would usually be determined through elicitation based on the distribution function of *W*. The distribution function for *W* when using the power prior Bayes factor (PPBF) is given by

(4) $$F\left(W\right)=\frac{{\left(V+W\right)}^{k+1}-{\left(V+a\right)}^{k+1}}{{\left(V+b\right)}^{k+1}-{\left(V+a\right)}^{k+1}}\;a\leq W\leq b.$$

So if a single percentile (*p*_1_) of the distribution of *W* is elicited (*w*_1_) then we find *k* by equating Equation (4) to *p*_1_ with *W* replaced by *w*_1_. For the PPBF and exponential prior Bayes factor (EPBF) this can easily be solved algebraically. The hybrid prior Bayes factor (HPBF) requires some other numerical search method. The distribution functions for all four priors are given in Table 2. A more reliable strategy is to determine suitable hyperparameters by optimizing the fit of multiple percentiles elicited from an expert. For example, if we elicit *h* percentiles $\left(p_{1},p_{2},...,p_{h}\right)$ of *W* $\left(w_{1},w_{2},...,w_{h}\right)$, then we find $\hat{k}$ such that

(5) k^= argmin k∑i=1h(V+wi)k+1−(V+a)k+1(V+b)k+1−(V+a)k+1−pi2.

Rather than directly eliciting *W* values, it is likely that an expert will find it easier to envisage particular central probability intervals (PIs) for the ORs. For the *i*th percentile to be elicited ($p_{i}$), let  PI u,i represent the upper limit of the $z_{i}$th central PI for the OR. Then $w_{i}$ can be found using wi=(ln( PI u,i)/Φ−1(pi))2, where $p_{i}=1-\left(1-0.01z_{i}\right)/2$. It is also likely to help if they are encouraged to choose the particular PIs $z=(z_{1},z_{2},...,z_{h})$ themselves.

**Table 2:** Prior distribution functions for *W*

Type of prior	F(W)	Limitations
Power	$\frac{{\left(V+W\right)}^{k+1}-{\left(V+a\right)}^{k+1}}{{\left(V+b\right)}^{k+1}-{\left(V+a\right)}^{k+1}}$	$k<-\frac{1}{2},k\neq -1$
	$\ln\left(\frac{V+W}{V+a}\right)/\ln\left(\frac{V+b}{V+a}\right)$	k=−1
Exponential	$\frac{\exp\left(-\frac{cW}{2}\right)-\exp\left(-\frac{ca}{2}\right)}{\exp\left(-\frac{cb}{2}\right)-\exp\left(-\frac{ca}{2}\right)}$	c>0
Hybrid	$\frac{\Gamma \left(-k-1,\frac{d}{2(V+W)}\right)-\Gamma \left(-k-1,\frac{d}{2(V+a)}\right)}{\Gamma \left(-k-1,\frac{d}{2(V+b)}\right)-\Gamma \left(-k-1,\frac{d}{2(V+a)}\right)}$	$d>-\hat{\beta_{1}},k<-1$
Reciprocal	$\frac{\ln\left(\frac{W+V}{a+V}\right)+\sum_{n=1}^{\infty }\frac{{\left(-1\right)}^{n}}{nn!}\left({\left(\frac{W+V}{2}\right)}^{n}-{\left(\frac{a+V}{2}\right)}^{n}\right)}{\ln\left(\frac{b+V}{a+V}\right)+\sum_{n=1}^{\infty }\frac{{\left(-1\right)}^{n}}{nn!}\left({\left(\frac{b+V}{2}\right)}^{n}-{\left(\frac{a+V}{2}\right)}^{n}\right)}$	

Distribution functions for each of the four prior forms.

To calculate $\hat{k}$, we also need to specify *a*, *b*, and *V*. Suppose the expert provides the minimum and maximum values of the upper limits of say the 80th percentile (denoted  PI u,min and  PI u,max) that they consider plausible. We can use  PI u,min to determine *a* using a=(ln( PI u,min)/Φ−1(p))2 where in this case z=60,p=0.8 and similarly for *b* replacing  PI u,min with  PI u,max. The value of *V* will be different for every SNP, so we suggest taking the median of the range of *V*. The values of *V* can be found by fitting univariate logistic regression models to the data where *V* is the square of the standard error of the parameter estimate for the SNP. This can be done in many standard statistical software packages.

The values of the hyperparameters in Figure 1 give a good indication of the space over which to search. We have written **R** code to carry out this search over the hyperparameters. The output includes the minimum sum of squares from Equation (5), so that the form of the prior which results in the smallest value can be determined. This code is available upon request. There are other methods available to determine the hyperparameters, for example, empirical Bayes methods. In empirical Bayes, the hyperparameters (Λ) are found as the solution to  argmax Λ(p(data|Λ)). In our case, this corresponds to maximizing the BF over Λ, which cannot be done analytically.

### Testing the Properties and Efficacy of the New BFs on Simulated Data

Three of out four forms of BF use the genotype data to inform the prior through *V*, the asymptotic variance of the estimate of the logOR. *V* will be different for each SNP since it depends on, among other quantities, the MAF. We used simulated data to generate realistic values of *V* and examined their effect on the prior density of *W* for values of *V* corresponding to SNPs that have an MAF not less than 0.005, as these are the SNPs that we might have sufficient power to detect with current sample sizes. These datasets were simulated using hapgen2 [Spencer et al., 2009] and the European haplotypes of the August 2010 release of the 1,000 genomes data with large sample sizes reflecting those now being generated by disease‐specific consortia.

There has been some suggestion that the effect size of causal SNPs may increase with decreasing MAF [Wang et al., 2005]. We investigate whether the three empirical forms of prior implicitly have this property. To assess this we examine how E(W) changes with *V*, over a support relevant to studies with sample sizes of 2,000 or more. Since SNPs with lower MAFs have larger *V* [Slager and Schaid, 2001], an appropriate prior would possess the property that E(W) is a nondecreasing function of *V*. Then as the MAF decreases, *V* increases and rarer SNPs have a priori larger effects on average.

We also tested the use of the novels BFs as a method for filtering (narrowing down the set of candidate causal variables) in a fine‐mapping study by carrying out such an analysis on simulated datasets with known causal SNPs. We give results for scenarios in which the causal SNP has an MAF of 0.08, for ORs of 1.10, 1.14, and 1.18 and for total sample sizes of 2,000, 4,000, and 20,000 (with an equal number of cases and controls). We simulated 1,000 datasets for this scenario, and illustrate the results using receiver operating characteristic (ROC) curves. Fawcett [2006] outlines several ways to determine ROC curves when they are used to represent a summary of multiple analyses (in this case those on each of the datasets). Our ROC curves present the true and mean false‐positive rates (FPRs) over the multiple analyses, but give no indication as to the variation in FPRs between datasets. Fawcett calls the method that we use threshold averaging.

### Comparing Fine‐Mapping Methods for the *CASP8* Region Using iCOGS Data

The Collaborative Oncological Gene‐environment Study (COGS) Consortium have recently carried out a number of studies using a specially developed Illumina array, known as the iCOGS array [Michailidou et al., 2013]. This was designed to fine‐map regions that had been previously identified by GWAS, by concentrating a large number of SNPs in regions of interest where there is already thought to be a causal association with breast, ovarian, or prostate cancer. One such region comprises base positions 201500074–202569992 of chromosome two, including the *CASP8* gene. In this region, 585 SNPs were originally genotyped on breast cancer case and control samples from the Breast Cancer Association Consortium and 501 passed quality control checks. A further 1,232 were successfully imputed using impute2 (Marchini and Howie, 2010), resulting in genotypes for 1,733 SNPs in 46,450 cases and 42,600 controls (total sample size: 89,050). We used both the full dataset and a subset of 5,238 individuals (2,721 cases and 2,517 controls) to assess the impact of our priors on both smaller and larger studies.

Prior to receiving the data, we carried out elicitation with a breast cancer genetics expert who had previously been involved in studies into the *CASP8* region. We then determined the prior distribution that best matched their beliefs and used it to calculate BFs and carry out filtering on the genotype data from iCOGS.

## Results

### The Dependence of the Prior Densities of *W* upon Hyperparameters and the Genotype Data

Figure 1 shows some possible prior densities for *W*, with V=0.003 for those densities which depend on *V*. We see that most of the priors put the majority of the weight of *W* close to the lower limit of its support (in this case close to zero). Other than being independent of the genotype data, one of the main advantages of the exponential prior over the other forms for f(W) is its ability to provide an almost uniform prior distribution for *W* over the range of values likely to be considered appropriate. The HPBF allows for more flexibility in the shape of the prior distribution. In particular, it is the only prior that allows more mass at higher values of *W* than at lower values of *W* and the only prior to have a stationary point. While the other priors are monotonically decreasing with *W*, the mode of hybrid prior is at W=−(V+d/2k).

**Figure 2:**
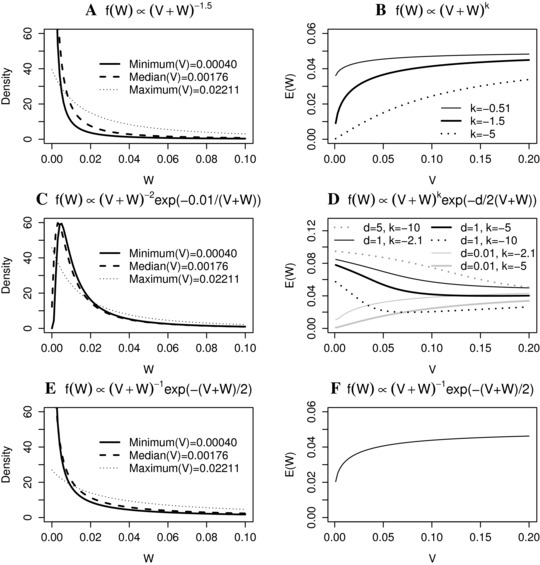
Prior densities of *W* (plots (a), (c), and (e)) and E(W) as a function of *V* (plots (b), (d), and (f)) for empirical forms of the prior (0<W≤0.1). Prior densities are given for minimum, median, and maximum values of *V* for SNPs with MAF>0.005 in a sample size of 20,000. E(W) is given over a range of *V* likely to been seen in sample sizes of 2,000 or greater with different values of the hyperparameters, where relevant.

To assess the impact of *V* on our priors in an intermediate sized fine‐mapping study, we simulated a dataset of size 20,000 (using exactly the same scenario as for the simulated dataset of 4,000) and determined the distribution of values of *V* for those SNPs with MAF ⩾0.005 and then determined the minimum, median, and maximum of these values to be 0.00040, 0.00176, and 0.02211, respectively. Figure 2 panels (a), (c), and (e) show the prior densities for *W* for the power, hybrid, and reciprocal priors for these three values of *V*, using fixed values of the hyperparameters. While there is some variation in the prior for *W* as *V* takes its maximum and minimum values, for most of the SNPs with MAF ⩾0.005 the prior will be relatively similar. There is little reason to suspect these observations will not generalize to other genomic regions.

We next consider SNPs with extreme values of *V* as these may lead to extreme priors and potentially large BFs. Because *V* is bounded below by 0, we only need to consider extreme large values of *V*. If the number of cases and controls is equal and denoted by *n*, Slager and Schaid [2001] showed that V∝1/n approximately. Studies with total sample sizes of 2,000 will yield most values of *V* in the region 0.005≤V≤0.2 for SNPs with MAF greater than 0.005. As sample size decreases occasional large values of *V* may be seen resulting from rare alleles (with MAFs less than 0.005) having very low counts in the case and/or control groups. These SNPs will have broad likelihoods and so are unlikely to have high BFs regardless of the prior. So although very rare alleles may have priors that are different from most of the SNPs, these rare SNPs are unlikely to be retained after filtering.

### Are These Priors Consistent with Rare Alleles Having Larger Effects?

There is some suggestion in the literature that rare SNPs might be expected to have larger effect sizes [Wang et al., 2005]. We wanted to assess whether this was the case for our suggested priors. To do this we examined how the expected value of *W* varied with *V*. If the hypothesis is true then rarer alleles should have larger effect sizes, hence larger values of *W*. Rarer alleles also have larger values of *V*. So if our priors possess this property, we should observe that E(W) increases with *V*. For the power, hybrid, and reciprocal priors, the expected value of *W* can either be found directly using integration by parts or by finding E(V+W) by integration and then using the relationship E(W)=E(V+W)−V. Those expectations that depend on *V* are plotted in Figure 2 panels (b), (d), and (f) for values of *V* likely to occur. E(W) is independent of *V* for the exponential prior. For the three expectations which depend on *V*, we were unable to verify algebraically that dE(W)/dV>0∀V>0,b>a>0andd,k within the specified limits (where relevant), but Figure 2 plots E(W) against *V* for a range of values of the hyperparameters. Panels (b) and (f) show this appears to be the case for the power and reciprocal prior. However, we can see from panel (d) of Figure 2 that E(W) could decrease with *V* for the hybrid prior. Researchers who believe that rare SNPs will a priori have larger effects should constrain *d* to be close to zero. Alternatively a generalization of the Savage‐Dickey density ratio (Verdinelli and Wasserman, 1995) could be used with a prior of the form $W^{k}\exp\left(-d/2W\right)$, so removing the dependence on *V*. The generalization of the Savage‐Dickey density ratio approximates the BF without the need for integration, hence allowing for a wider range of priors. It bases calculation of the BF on a large number of samples from the posterior distributions of the model parameters.

### Fine‐Mapping Using BFs on Simulated Data

We carried out filtering using WBFs and our new BFs on 1,000 simulated datasets with a single causal SNP with an MAF of 0.08. We chose ORs of 1.10, 1.14, and 1.18 and sample sizes of 2,000, 4,000, and 20,000. We also included the results of filtering using *P*‐values from univariate logistic regression. Table 3 shows the true‐positive rates (TPRs) (× 1, 000) from the simulated data analysis by sample size, OR, and FPR. We have limited the results to FPRs ≤20% as this more than covers the desired range of FPRs in any fine‐mapping procedure. The support for the new priors is 0.003≤W≤0.1 and so we let W=0.003 and W=0.1 in the WBF in Table 3. In Table 3, we chose hyperparameters for our new priors that place more of the mass at smaller values of *W*. The priors for PPBF and HPBF with the specified hyperparameters have similar shaped densities and so, not surprisingly, produce broadly similar rankings.

**Table 3:** TPRs (× 1, 000) from the simulated data analysis by sample size, OR, and FPR

	Sample size (SS)											
	SS = 2,000				SS = 4,000				SS = 20,000			
	FPR (%)				FPR (%)				FPR (%)			
Method (parameter values)	5%	10%	15%	20%	5%	10%	15%	20%	1%	5%	10%	15%
	OR = 1.10											
*P*‐value	72	118	163	259	151	249	320	384	202	497	677	786
WBF (W=0.003)	48	**131**	**216**	**291**	136	**285**	**385**	**442**	**231**	**579**	**762**	**836**
WBF (W=0.1)	**89**	**126**	**166**	173	**152**	221	242	259	173	433	530	582
PPBF (k=−1.5)	**88**	**140**	**202**	245	**170**	**264**	318	346	**224**	**524**	676	758
HPBF (d=0.01,	**89**	**140**	**204**	248	**170**	**263**	308	334	**225**	**517**	655	707
k=−1.1)												
	OR = 1.14											
*P*‐value	146	247	331	395	232	357	457	531	508	831	924	962
WBF (W=0.003)	106	**255**	**376**	**457**	222	**408**	**528**	**574**	502	**850**	**960**	**979**
WBF (W=0.1)	**159**	**248**	290	307	**239**	335	373	387	503	789	875	899
PPBF (k=−1.5)	**159**	**272**	**331**	372	**257**	**386**	**457**	486	**536**	**846**	**926**	956
HPBF (d=0.01,	**161**	**276**	**332**	363	**256**	**387**	450	470	**539**	**843**	919	943
k=−1.1)												
	OR = 1.18											
*P*‐value	178	286	382	454	323	475	551	619	753	950	984	994
WBF (W=0.003)	134	**302**	**430**	**496**	290	**500**	**614**	**663**	730	**950**	**989**	**997**
WBF (W=0.1)	**200**	**298**	340	353	**328**	452	478	495	751	947	980	988
PPBF (k=−1.5)	**196**	**322**	**397**	439	**351**	**496**	**560**	602	**767**	**954**	**987**	**994**
HPBF (d=0.01,	**207**	**325**	**396**	434	**355**	**493**	**551**	582	**770**	**955**	**987**	**994**
k=−1.1)												

True‐positive rates (TPRs) multiplied by a thousand at the most relevant false‐positive rates (FPRs) for different filtering methods (PPBF, HPBF, Wakefield Bayes factors, and *P*‐values) applied to 1,000 simulated datasets with 2,871 SNPs. For PPBF and HPBF, the support is 0.003≤W≤0.1. The data were simulated using the LD structure of the *CASP8* region for a scenario with a single causal SNP with an MAF of 0.08 for various sample sizes, odds ratios, and FPRs . Figures in bold are those that exceed the TPR obtained using *P*‐values.

Table 3 shows that using WBF with W=0.003 yields higher TPRs than using WBF with W=0.1 in many of the scenarios considered. This is perhaps not surprising because the logORs for the causal SNP used in the simulations are all small (0.095–0.166) and so lower values of *W*, which put more weight at small ORs, are expected to perform better. The exception to this is at the lowest FPR where W=0.1 generally yields higher TPRs. It is at the very low (and arguably most relevant) FPRs that the PPBF and HPBF methods generally outperform all the other methods considered. If we focus on the smallest FPR considered at each level of sample size and OR, we see that either the HPBF or the PPBF have the highest TPR of any of the methods considered in eight out of the nine scenarios. It is only when the sample size is 20,000 and the OR is 1.1 that one of the other methods (WBF with W=0.003) is superior at the lowest FPR.

What is somewhat surprising is that even when the sample size is as high as 20,000, there are substantial differences in the performances of the *P*‐value method and the methods using BFs, For example, with a sample size of 20,000, an OR of 1.10 and an FPR of 5%, the TPRs for the *P*‐value and WBF with W=0.003 are 0.497 and 0.579, respectively. The TPR for the WBF method is nearly 17% bigger than the TPR for the *P*‐value method. Making the same comparison when the FPR is 1% the WBF method is over 14% bigger than the TPR for the *P*‐value (0.231 compared to 0.202).

**Figure 3:**
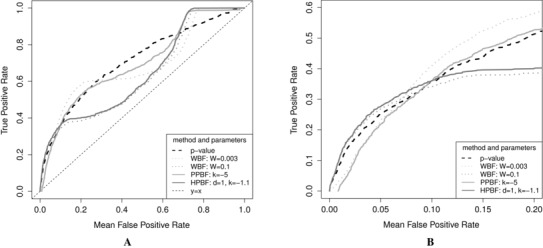
ROC curves showing the results of WBF, PPBF, HPBF, and *P*‐value filtering. (A) Shows the whole ROC space while (B) shows the ROC curve for false‐positive rates below 20%. We use W=0.003 and W=0.1 for the WBF analysis. The prior for the PPBF analysis has k=−5 and puts most of the mass close to W=0.003. The prior for the HPBF analysis has d=1 and k=−1.1 and puts most of the weight close to W=0.1. All filtering was carried out on 1,000 datasets simulated using the LD structure of the *CASP8* region for a scenario with a single causal SNP that has an OR of 1.14, an MAF of 0.08, and a total sample size of 4,000.

Figure 3 shows several ROC curves derived from filtering with a causal SNP with an OR of 1.14, an MAF of 0.08 and a sample size of 4,000. Figure 3a shows the whole ROC space while Figure 3b shows the ROC curve for FPRs below 20%. Figure 3 includes the results of filtering using the WBF approximation with W=0.003 and W=0.1 and also the results of filtering using a power prior and a hybrid prior with support 0.003≤W≤0.1. The two new priors were chosen so that the power prior put a lot of mass at W=0.003 and hybrid prior out a lot of mass around W=0.1. Consequently, using the power prior produces an ROC curve similar to that using WBFs with W=0.01 and using the hybrid prior produces an ROC curve similar to WBF with W=0.01. There is clearly a lot of variation in the effectiveness of the WBF filter as *W* changes. Putting too much prior weight on large effect sizes clearly leads to poor performance of the WBF when the actual causal effect size is small. The new BFs can be thought of as a weighted average of the BFs over the support of *W* and so do not suffer to the same degree as using WBF with a value of *W* that puts a lot of mass at values of β that have a low likelihood.

### Fine‐Mapping Using BFs on the iCOGS Data

Before analyzing the iCOGS data [Michailidou et al., 2013], we carried out elicitation with a breast cancer genetics expert. They believed that there was a causal SNP with a small effect size in the *CASP8* region on chromosome two. We initially asked our expert to give an interval of possible ORs at a single percentile. They thought that the 80th percentile of the OR was between 1.05 and 1.3, yielding a=0.003 and b=0.1. We then asked them to choose three further PIs that they were comfortable thinking in terms of. They provided the 0.95, 0.75, and 0.50 PIs giving $\left(z_{1},\;z_{2},\;z_{3}\right)=\left(95,\;75,\;50\right)$. For these probabilities, we asked for their best estimate of the upper limit of the OR and the expert provided  PI u=(1.43,1.21,1.14). From these we get $\left(w_{1},\;w_{2},\;w_{3}\right)=\left(0.0333,0.0275,0.0377\right)$. The median value of *V* for the SNPs in the iCOGS data was 0.00017. We used all these values to carry out a search over −10≤k<−0.5 at intervals of 0.01 for the PPBF. Evaluating Equation (5) for each of these values of *k*, we found that for the PPBF, the minimum of the sum of squared differences occurs at k=−1.66. If we use the same method to find other prior forms which fit the elicited values we get an exponential prior with c=145 and a hybrid prior with d=0.001 and k=−1.69. Of these three priors, the power prior has the closest fit to the elicited values and is a better fit than the reciprocal prior, so we used this to calculate the BF for the iCOGS data.

Table 4 contains information about the top 20 ranked SNPs based on the PPBF values calculated from the full iCOGS data. It should be noted that none of these will necessarily be the causal SNP, although previous simulations show us that, with such a large sample size, there is a high probability that the causal SNP will be highly ranked (Spencer et al., 2014). We observe that nine of the top 20 ranked SNPs were among the 501 SNPs genotyped (rather than imputed) in the iCOGS data. Included in the table is the ranking for these SNPs by *P*‐value from univariate logistic regression and by WBF with the two values of *W* that are the extremities of the support used for *W* with the PPBF. We can see that while all these methods rank SNPs similarly, with the same SNPs always ranked in the top three, the rankings do differ somewhat.

**Table 4:** Results of analysis of iCOGS data

				Ranking			
					WBF with W=		
SNP number	OR (95% CI)	MAF	PPBF	*P*‐value	0.003	0.1	PPBF
980^b^	1.048 (1.027, 1.071)	0.294	1,387	1	1	1	1
1,027	1.046 (1.024, 1.068)	0.285	664	2	2	2	2
992^b^	1.045 (1.022, 1.067)	0.287	334	3	3	3	3
909	1.043 (1.021, 1.065)	0.287	234	9	4	6	4
878^a^	1.081 (1.039, 1.125)	0.061	228	12	13	4	5
1,272^a^	1.075 (1.036, 1.116)	0.071	217	14	11	5	6
950^b^	1.043 (1.021, 1.065)	0.286	217	10	5	7	7
838	1.041 (1.020, 1.062)	0.338	213	5	6	10	8
960^b^	1.043 (1.021, 1.065)	0.285	213	7	=7	=8	=9
961^b^	1.043 (1.021, 1.065)	0.285	213	8	=7	=8	=9
985^b^	1.043 (1.021, 1.066)	0.286	206	4	9	11	11
837	1.042 (1.021, 1.064)	0.299	200	6	10	13	12
907	1.042 (1.020, 1.064)	0.287	167	11	12	16	13
896	1.042 (1.020, 1.064)	0.287	166	13	=14	=17	=14
912	1.042 (1.020, 1.064)	0.287	166	15	=14	=17	=14
956^a, b^	1.052 (1.025, 1.080)	0.170	159	16	16	15	16
681^a^	1.074 (1.035, 1.116)	0.069	149	17	19	14	17
1,004^a, b^	1.051 (1.024, 1.078)	0.173	124	18	18	20	18
885	1.041 (1.019 1.063)	0.287	119	19	17	23	19
955^a, b^	1.050 (1.023, 1.078)	0.173	112	21	21	21	20

Notes

For these SNPs, the major allele is associated with a higher disease risk.

These SNPs were not genotyped but imputed. Top‐ranked SNPs in *CASP8* region based on power prior Bayes factor (PPBF) approximation with hyperparameter k=−1.66 and a=0.003≤W≤b=0.1. Rankings using *P*‐value and Wakefield Bayes factor (WBF) are also included, as is the logistic regression estimate and 95% confidence interval (CI) of the odds ratio (OR) for each SNP. The genotype data for *CASP8* region come from the iCOGS study and has a total sample size of 89,050 and 1,733 SNPs.

With such a large sample size, the majority of the information comes from the likelihood, rather than the prior, which is why the rankings using the different methods are so similar when considering the full iCOGS data. Many association studies will have smaller sample sizes than those used here and in these studies the prior will have more influence on the BFs obtained and so we would expect much more variation in the ranks across the different BFs. We investigated this using a stratified random subset of the iCOGS subjects, with 2,721 cases and 2,517 controls (5,238 total). The same analyses were carried out on this subset of the data and the results are given in Table 5. As expected, the prior has much larger influence in this smaller fine‐mapping study. Of the top 20 SNPs selected by PPBF, only four of these are in the top 20 for WBF with W=0.003 (where the prior has most mass around small effect sizes). Of the top 20 SNPs selected by WBF with W=0.003, 10 SNPs are not in the top 50 for PPBF and three are not in the top 75 for PPBF. There is more agreement between WBF with W=0.1 and PPBF but WBF still selects two SNPs in its top 20 that are not in the top 20 for PPBF.

**Table 5:** Results of subset analysis of iCOGS data

				Ranking			
					WBF with W=		
SNP number	OR (95% CI)	MAF	PPBF value	*P*‐value	0.003	0.1	PPBF
822^a^	1.514 (1.215, 1.886)	0.037	45.1	1	28	1	1
807^a^	1.520 (1.216, 1.900)	0.036	42.2	2	35	2	2
820^a^	1.515 (1.213, 1.893)	0.036	40.0	3	37	3	3
824^a^	1.514 (1.212, 1.891)	0.036	39.7	4	39	4	4
868^a^	1.508 (1.209, 1.881)	0.038	38.5	5	38	5	5
378^a^	1.431 (1.174, 1.745)	0.046	33.3	7	16	6	6
858^a^	1.495 (1.198, 1.866)	0.036	30.6	6	47	7	7
379^a^	1.409 (1.162, 1.709)	0.047	29.2	8	15	8	8
854	1.470 (1.181, 1.829)	0.036	24.0	9	56	9	9
346	1.262 (1.099, 1.449)	0.093	23.7	22	2	27	10
845^a^	1.469 (1.180, 1.829)	0.037	23.6	11	57	10	11
879^a^	1.480 (1.184, 1.851)	0.037	23.2	10	64	11	12
823^a^	1.480 (1.183, 1.851)	0.036	22.9	12	65	12	13
339^a^	1.266 (1.099, 1.459)	0.091	21.7	28	3	37	14
705^a^	1.439 (1.161, 1.761)	0.043	20.5	15	53	15	15
752^a^	1.449 (1.168, 1.798)	0.039	20.2	14	61	14	16
900^a^	1.475 (1.177, 1.849)	0.036	19.7	13	89	13	17
698^a^	1.454 (1.167, 1.812)	0.036	18.3	16	79	16	18
699^a^	1.454 (1.167, 1.812)	0.036	18.3	17	80	17	19
700^a^	1.432 (1.159, 1.771)	0.038	18.2	20	63	19	20

Notes

These SNPs were imputed. Ranks of the top‐ranked SNPs in *CASP8* region based on the power prior Bayes factor (PPBF) approximation with hyperparameter k=−1.96 and a=0.003≤W≤b=0.1. Rankings using *P*‐value and Wakefield Bayes factor (WBF) are also included, as is the logistic regression estimate and 95% confidence interval (CI) of the odds ratio (OR) for each SNP. Values of the Bayes factors for the PPBF are also provided. The genotype data for *CASP8* region come from a subset of the iCOGS study and has a total sample size of 5,238 and 1,733 SNPs.

## Discussion

### Novel BFs and Their Properties

We have developed several new forms of approximate BF. These include three parametric families and one fixed form, each relating to a different prior distribution on *W*, where we assume that the prior distribution on the logOR of an SNP is N(0,W). This allows for the calculation of BFs where a normal distribution is believed to be an appropriate form for the prior, but where there is uncertainty in *W*. Most of the priors we suggest for *W* put most of the weight of *W* at the lower end of the support but the exponential prior allows for an almost uniform prior which is useful when an expert believes a range of values of *W* are equally likely a priori. The hybrid prior can be specified so that the mode is anywhere in the given range. This might be useful when an expert has a strong prior belief in a particular value of *W* but wants to allow for some uncertainty in it.

Three of these prior forms also depend upon the genotype data. The expectation of *W* appears universally to be an increasing function of *V* only for the power and reciprocal priors. This means that these priors are consistent with the hypothesis that rarer alleles have have larger effects. Depending on the values of the hyperparameters, E(W) may be a decreasing function of *W* when the hybrid prior is employed. Such a prior would be inappropriate to use if it was believed that rare causal SNPs do indeed have larger effect sizes. In this case, we suggest a Savage‐Dickey density ratio approach [Verdinelli and Wasserman, 1995].

All the novel BFs described here are univariate. The solution we have employed in the univariate case relies on recognizing the BF integrands as standard probability densities. We did consider extending these BFs to the multivariate case and while specifying the prior in the multivariate case is straightforward, the resulting integrals appear to be intractable and would probably need to be solved analytically so that easily calculated closed form expressions for the BFs may not be easily available.

### Using Novel BFs in Practice

All novel BF approximations can be calculated in **R** (we give code in supplementary data), although the EPBF is computationally intensive and cannot produce results for SNPs which have very small MLEs of the logOR. The computation for all of the other forms is simple and efficient and we therefore recommend using the PPBF or HPBF instead of the EPBF where possible. In most cases, hyperparameters can be found which result in power or hybrid priors very similar to the desired exponential prior. In these situations, we recommend that the EPBF only be used if the investigator does not want to include any information from the data in the prior.

We described how elicitation may be employed with an expert to determine the most appropriate values for the hyperparameters, although such experts may find this task difficult. The importance of feedback in the elicitation process is worth emphasizing. Once a distribution for *W* has been determined based on the quantiles elicited from the expert, it is important to relay back to them what this means about other quantiles not elicited to check that these are acceptable. A web‐based tool, MATCH, which may help with this purpose is now available [Morris et al., 2014].

### BF Analysis in Future Genetics

Filtering using BFs has already been used in fine‐mapping [Maller et al., 2012] and our more flexible approach is likely to appeal to investigators who struggle to specify a suitable variance for a fixed normal prior. The methods we use assume that all SNPs have equal prior odds of being causal, but BFs can also be used to update the odds and then filtering can be carried out using the posterior odds of causality. The effectiveness of BF filtering may be further improved by appropriate incorporation of functional information through SNP‐specific prior odds. Such functional data can be found on the encode database [Encode Project Consortium, 2011], the RegulomeDB database [Boyle et al., 2012] and the F‐SNP database [Lee and Shatkay, 2009]. All functional SNP‐level data sources are currently limited as information is not complete for all the SNPs across the genome. The FS score found on the F‐SNP database has the advantage that it integrates a large amount of data from multiple publicly available data sources. It formally combines scores from a number of bioinformatics tools using weighting based on the “reliability” of these tools to give a score between 0 and 1. BFs are likely to become increasingly popular as investigators seek to make use of the vast prior functional information available. The new BFs presented in this paper allow researchers more flexibility in the specification of the prior OR, allowing the distribution used to better match expert prior beliefs.

## Supporting information

Disclaimer: Supplementary materials have been peer‐reviewed but not copyedited.

Supplementary material.
