# Supplementary material for: Novel Bayes Factors That Capture Expert Uncertainty in Prior Density Specification in Genetic Association Studies
Source: Genet Epidemiol. 2015 Feb 27;39(4):239–48. doi: 10.1002/gepi.21891 (PMC4406822; doi:10.1002/gepi.21891)
Supplement: Supplementary file 1 — Supplementary material. [file GEPI-39-239-s001.zip › gepi21891-sup-0001-text.pdf]

1 Derivation of novel Bayes factors

Wakefield's Bayes factor approximation (Wakefield, 2009) uses the prior $\beta_1 \sim N(0, W)$ for the logOR of the causal SNP and the asymptotic distribution $\widehat{\beta_1} \sim N(\beta_1, V)$ as the 'data'. We have modified this approximation to allow for uncertainty in W by putting various priors on it. Our PPBF, EPBF, HPBF and RPBF approximations are derived below. In all cases $0 < a \leq W \leq b$.

1.1 Deriving the Bayes factor when $f(W) = q(V + W)^k$ for $k \leq -1/2$

The power prior Bayes factor (PPBF) approximation is given by

$$\begin{aligned}\text{PPBF} =& \frac{\int_W \int_{\beta_1} p(\widehat{\beta_1}|\beta_1) f(\beta_1|W) f(W)\, d\beta_1\, dW}{p(\widehat{\beta_1}|\beta_1 = 0)} \\ =& \frac{1}{Q} \int_W \int_{\beta_1} \frac{1}{\sqrt{2\pi V}} \exp\left(-\frac{(\widehat{\beta_1} - \beta_1)^2}{2V}\right) \frac{1}{\sqrt{2\pi W}} \exp\left(-\frac{\beta_1^2}{2W}\right) q(V + W)^k\, d\beta_1\, dW, \quad (1)\end{aligned}$$

where

$$q = \begin{cases} (k+1)[(V+b)^{k+1} - (V+a)^{k+1}]^{-1} & k \neq -1 \\ [\ln(V+b) - \ln(V+a)]^{-1} & k = -1 \end{cases}$$

is the normalising constant and $Q = p(\widehat{\beta_1}|\beta_1 = 0) = \frac{1}{\sqrt{2\pi V}} \exp\left(-\frac{\widehat{\beta_1}^2}{2V}\right)$. Since neither limit depends on either W or β_1 we can choose the order of integration. Integrating out β_1 by rearranging the integrand into the density of a normal distribution in β_1 gives

$$\begin{aligned}\text{PPBF} &= \frac{q}{Q} \int_W \frac{1}{\sqrt{2\pi(V + W)}} \exp\left(-\frac{\widehat{\beta_1}^2}{2(V + W)}\right) (V + W)^k\, dW \\ &= \frac{q}{Q\sqrt{2\pi}} \int_W (V + W)^{k - \frac{1}{2}} \exp\left(-\frac{\widehat{\beta_1}^2}{2(V + W)}\right) dW. \quad (2)\end{aligned}$$

With $k < -\frac{1}{2}$ the integrand above takes the form of an inverse gamma density $f(y; \alpha, \gamma) = \frac{\gamma^\alpha}{\Gamma(\alpha)} y^{-(\alpha+1)} \exp\left(-\frac{\gamma}{y}\right)$ with shape and scale parameters $-(k + \frac{1}{2})$ and $\widehat{\beta_1}^2/2$, respectively.

$$
\begin{aligned}
\text{PPBF} =& \frac{q}{Q\sqrt{2\pi}} \frac{\Gamma(-k-\frac{1}{2})}{(\widehat{\beta_1}^2/2)^{-k-\frac{1}{2}}} \int_{W=a}^{W=b} \frac{(\widehat{\beta_1}^2/2)^{-k-\frac{1}{2}}}{\Gamma(-k-\frac{1}{2})} (V+W)^{k-\frac{1}{2}} \exp\left(-\frac{\widehat{\beta_1}^2}{2(V+W)}\right) dW \\
=& \frac{q}{Q\sqrt{2\pi}} \frac{\Gamma(-k-\frac{1}{2})}{(\widehat{\beta_1}^2/2)^{-k-\frac{1}{2}}} \int_{W=a+V}^{W=b+V} \frac{(\widehat{\beta_1}^2/2)^{-k-\frac{1}{2}}}{\Gamma(-k-\frac{1}{2})} W^{k-\frac{1}{2}} \exp\left(-\frac{\widehat{\beta_1}^2}{2W}\right) dW \\
=& \frac{q}{Q\sqrt{2\pi}} \frac{\Gamma(-k-\frac{1}{2})}{(\widehat{\beta_1}^2/2)^{-k-\frac{1}{2}}} \left[\frac{\Gamma(-k-\frac{1}{2}, \frac{\widehat{\beta_1}^2}{2W})}{\Gamma(-k-\frac{1}{2})}\right]_{W=a+V}^{W=b+V}.
\end{aligned}
$$

Using the form for Q and the normalising constant q given above yields

$$
\text{PPBF} = \begin{cases}
\dfrac{(k+1)\sqrt{V}\left[\Gamma\left(-k-\frac{1}{2}, \frac{\widehat{\beta_1}^2}{2(b+V)}\right) - \Gamma\left(-k-\frac{1}{2}, \frac{\widehat{\beta_1}^2}{2(a+V)}\right)\right]}{\left(\widehat{\beta_1}^2/2\right)^{-k-\frac{1}{2}} \exp\left(-\frac{\widehat{\beta_1}^2}{2V}\right)\left[(V+b)^{k+1} - (V+a)^{k+1}\right]} & k \neq -1 \\
\\
\dfrac{\sqrt{2V}\left[\Gamma\left(\frac{1}{2}, \frac{\widehat{\beta_1}^2}{2(b+V)}\right) - \Gamma\left(\frac{1}{2}, \frac{\widehat{\beta_1}^2}{2(a+V)}\right)\right]}{\widehat{\beta_1} \exp\left(-\frac{\widehat{\beta_1}^2}{2V}\right) \ln\left(\frac{V+b}{V+a}\right)} & k = -1
\end{cases}
$$

where $\Gamma(s,x) = \int_x^\infty t^{s-1} \exp(-t)\, dt$ represents the upper incomplete gamma function.

1.2 Deriving the Bayes factor when $f(W) = r \exp\left(-\frac{cW}{2}\right)$ for $c > 0$

For the exponential prior the normalising constant is $r = \frac{c}{2}\left[\exp\left(-\frac{ca}{2}\right) - \exp\left(-\frac{cb}{2}\right)\right]^{-1}$. For the EPBF, the steps used to get to Equation (2) for the PPBF are the same. So with the exponential prior we have

$$
\text{EPBF} = \frac{r}{Q} \int_a^b \frac{1}{\sqrt{2\pi(V+W)}} \exp\left(-\frac{\widehat{\beta_1}^2}{2(V+W)}\right) \exp\left(-\frac{cW}{2}\right) dW
$$

with Q defined in Appendix 1.1. Re-writing the integrand in the form of a generalized inverse Gaussian distribution with parameters $c > 0$, $\widehat{\beta_1}^2$ and $\frac{1}{2}$, we have

$$\text{EPBF} = \frac{2r\exp(\frac{cV}{2})K_{\frac{1}{2}}\left(\sqrt{c\widehat{\beta_1}^2}\right)}{Q\sqrt{2\pi}(c/\widehat{\beta_1}^2)^{\frac{1}{4}}} \int_{W=a}^{W=b} \frac{(c/\widehat{\beta_1}^2)^{\frac{1}{4}}(V+W)^{-\frac{1}{2}}}{2K_{\frac{1}{2}}\left(\sqrt{c\widehat{\beta_1}^2}\right)} \exp\left(-\frac{\widehat{\beta_1}^2}{2(V+W)} - \frac{c(V+W)}{2}\right) dW$$

$$= \frac{2r\exp(\frac{cV}{2})K_{\frac{1}{2}}\left(\sqrt{c\widehat{\beta_1}^2}\right)}{Q\sqrt{2\pi}(c/\widehat{\beta_1}^2)^{\frac{1}{4}}} \int_{W=a+V}^{W=b+V} \frac{(c/\widehat{\beta_1}^2)^{\frac{1}{4}}W^{-\frac{1}{2}}}{2K_{\frac{1}{2}}\left(\sqrt{c\widehat{\beta_1}^2}\right)} \exp\left(-\frac{\widehat{\beta_1}^2}{2W} - \frac{cW}{2}\right) dW$$

$$= \frac{2r\exp(\frac{cV^2+\widehat{\beta_1}^2}{2V})\sqrt{V}K_{\frac{1}{2}}\left(\sqrt{c\widehat{\beta_1}^2}\right)}{(c/\widehat{\beta_1}^2)^{\frac{1}{4}}} \int_{W=a+V}^{W=b+V} \frac{(c/\widehat{\beta_1}^2)^{\frac{1}{4}}W^{-\frac{1}{2}}}{2K_{\frac{1}{2}}\left(\sqrt{c\widehat{\beta_1}^2}\right)} \exp\left(-\frac{cW}{2} - \frac{\widehat{\beta_1}^2}{2W}\right) dW.$$

where $K_{\frac{1}{2}}(.)$ represents a modified Bessel function of the second kind. The integrand is a generalized inverse Gaussian density: $W \sim GIG(c, \widehat{\beta_1}^2, \frac{1}{2})$. Whilst there is no closed form expression for the distribution function it can be calculated in **R** using the `pgig` command.

1.3 Deriving the Bayes factor when $f(W) = s(V+W)^k \exp\left(-\frac{d}{2(V+W)}\right)$ for $d > -\widehat{\beta_1}^2$, $k < -1$

The hybrid prior takes the form of an inverse gamma density, therefore the normalising constant is $s = \left(\frac{d}{2}\right)^{-k-1}\left[\Gamma\left(-k-1, \frac{d}{2(b+V)}\right) - \Gamma\left(-k-1, \frac{d}{2(a+V)}\right)\right]^{-1}$. This applies only with $k < -1$.

Again following the steps that give us Equation (2) for the PPBF, we have this form for the HPBF:

$$\text{HPBF} = \frac{s}{Q\sqrt{2\pi}} \int_a^b (V+W)^{k-\frac{1}{2}} \exp\left(-\frac{\widehat{\beta_1}^2}{2(V+W)}\right) \exp\left(-\frac{d}{2(V+W)}\right) dW.$$

As with the PPBF, the integrand above takes the form of an inverse gamma density for the values of k we have specified. In this case the shape and scale parameters are $-(k+\frac{1}{2})$ and $(\widehat{\beta_1}^2 + d)/2$, respectively.

$$
\begin{aligned}
\text{HPBF} =& \frac{s}{Q\sqrt{2\pi}} \frac{\Gamma(-k-\frac{1}{2})}{((\widehat{\beta_1}^2+d)/2)^{-k-\frac{1}{2}}} \int_{W=a}^{W=b} \frac{((\widehat{\beta_1}^2+d)/2)^{-k-\frac{1}{2}}}{\Gamma(-k-\frac{1}{2})} (V+W)^{k-\frac{1}{2}} \exp\left(-\frac{(\widehat{\beta_1}^2+d)}{2(V+W)}\right) dW \\
=& \frac{s}{Q\sqrt{2\pi}} \frac{\Gamma(-k-\frac{1}{2})}{((\widehat{\beta_1}^2+d)/2)^{-k-\frac{1}{2}}} \int_{W=a+V}^{W=b+V} \frac{((\widehat{\beta_1}^2+d)/2)^{-k-\frac{1}{2}}}{\Gamma(-k-\frac{1}{2})} W^{k-\frac{1}{2}} \exp\left(-\frac{(\widehat{\beta_1}^2+d)}{2W}\right) dW \\
=& \frac{s}{Q\sqrt{2\pi}} \frac{\Gamma(-k-\frac{1}{2})}{((\widehat{\beta_1}^2+d)/2)^{-k-\frac{1}{2}}} \left[\frac{\Gamma(-k-\frac{1}{2}, \frac{(\widehat{\beta_1}^2+d)}{2W})}{\Gamma(-k-\frac{1}{2})}\right]_{W=a+V}^{W=b+V} \\
=& \frac{\sqrt{2V}\exp\left(\frac{\widehat{\beta_1}^2}{2V}\right)\left[\Gamma\left(-k-\frac{1}{2}, \frac{(\widehat{\beta_1}^2+d)}{2(b+V)}\right) - \Gamma\left(-k-\frac{1}{2}, \frac{(\widehat{\beta_1}^2+d)}{2(a+V)}\right)\right]}{(\widehat{\beta_1}^2+d)^{-k-\frac{1}{2}} d^{k+1}\left[\Gamma\left(-k-1, \frac{d}{2(b+V)}\right) - \Gamma\left(-k-1, \frac{d}{2(a+V)}\right)\right]}.
\end{aligned}
$$

1.4 Deriving the Bayes factor when $f(W) = \frac{t}{(V+W)} \exp\left(-\frac{(V+W)}{2}\right)$

The reciprocal prior approximate Bayes factor (RPBF) is a specific prior distribution with normalising constant $t = [\Gamma(0,(a+V)/2) - \Gamma(0,(b+V)/2)]^{-1}$. The form of the upper incomplete gamma function $\Gamma(0,z)$ is a special case and is calculated using the relationship $\Gamma(0,z) = -\text{Ei}(-z) = -\gamma - \ln(z) - \sum_{n=0}^{\infty}(-1)^n \frac{z^n}{nn!}$, where Ei is the exponential integral and γ is the Euler-Mascheroni constant.

The RPBF can be written

$$
\text{RPBF} = \frac{t\exp(-|\widehat{\beta_1}|)}{Q} \int_a^b \frac{1}{\sqrt{2\pi}(V+W)^{\frac{3}{2}}} \exp\left(-\frac{\widehat{\beta_1}^2}{2(V+W)}\right) \exp\left(-\frac{(V+W)-2|\widehat{\beta_1}|}{2}\right) dW,
$$

which can be further simplified by writing the integrand as the density of an inverse Gaussian distribution with mean and scale parameters of $|\widehat{\beta_1}|$ and $\widehat{\beta_1}^2$ respectively.

$$
\begin{aligned}
\text{RPBF} &= \frac{t\exp(-|\widehat{\beta_1}|)}{Q} \int_{W=a}^{W=b} \frac{1}{\sqrt{2\pi}(V+W)^{\frac{3}{2}}} \exp\left(-\frac{\left((V+W)-|\widehat{\beta_1}|\right)^2}{2(V+W)}\right) dW \\
&= \frac{t\exp(-|\widehat{\beta_1}|)}{Q|\widehat{\beta_1}|} \int_{W=a+V}^{W=b+V} \left[\frac{\widehat{\beta_1}^2}{2\pi W^3}\right]^{\frac{1}{2}} \exp\left(-\frac{\widehat{\beta_1}^2(W-|\widehat{\beta_1}|)^2}{2\widehat{\beta_1}^2 W}\right) dW \\
&= \frac{t\exp(-|\widehat{\beta_1}|)}{Q|\widehat{\beta_1}|} \left[\Phi_+(W) + \exp(2|\widehat{\beta_1}|)\Phi_-(W)\right]_{W=a}^{W=b},
\end{aligned}
$$

where

$$\Phi_+(y) = \Phi\left(\sqrt{y+V} - \frac{|\widehat{\beta_1}|}{\sqrt{y+V}}\right), \qquad \Phi_-(y) = \Phi\left(-\sqrt{y+V} - \frac{|\widehat{\beta_1}|}{\sqrt{y+V}}\right)$$

and $\Phi(.)$ is the distribution function of the standard normal distribution. So

$$\text{RPBF} = \frac{\sqrt{2\pi V}\exp\left(\frac{\widehat{\beta_1}^2}{2V} - |\widehat{\beta_1}|\right)\left[\Phi_+(b) - \Phi_+(a) + (\Phi_-(b) - \Phi_-(a))\exp(2|\widehat{\beta_1}|)\right]}{|\widehat{\beta_1}|\left[\ln\left(\frac{V+b}{V+a}\right) + \sum_{n=1}^{\infty}\frac{(-1)^n}{nn!}\left(\left(\frac{b+V}{2}\right)^n - \left(\frac{a+V}{2}\right)^n\right)\right]}.$$

2 `R` code to calculate the new Bayes factors

The **R** code given below will calculate a vector of approximate Bayes factors for a set of SNPs which have been genotyped and analysed using single SNP logistic regression models. These models should all include the same relevant covariates. The first two inputs are `betas` and `vars`, which are both vectors of length n, where n is the number of SNPs in the genotyping study. Respectively, they should be the fitted logOR estimates ($\widehat{\beta_1}$) and their variances (V) from the logistic regression models. The other inputs are `form`, which can be either `"PPBF"`, `"EPBF"`, `"HPBF"` or `"RPBF"` indicating the form of approximate BF to be used; `hyper`, indicating the values of the hyperparameters, which is a single value if `form="PPBF"` or `"EPBF"`, a vector of length 2 (`c(d,k)`) if `form="HPBF"` and null if `form="RPBF"`; `a` and `b`, the limits of the range ($a < b$) over which W should be defined.

```
BFapprox<-function(betas,vars,form,hyper,a=0.003,b=0.1){
nSNP<-length(betas)
betasq<-betas^ 2
#define Q, the denominator
Q=((2*pi*vars)^ -0.5)*exp(-betasq/(2*vars))
######################################################
switch(form,
#Power prior form
PPBF={
k=hyper
#normalising constant for prior
```

```
if(k!=-1){
q<-(k+1)/((vars+b)^(k+1)-(vars+a)^(k+1)) }
if(k==-1){
q<-(log(vars+b)-log(vars+a))^(-1) }
#BF
num<-gamma(-k-0.5)*(pgamma(betasq/(2*(vars+a)), -k-0.5)-pgamma(betasq/(2*(vars
+b)), -k-0.5))
denom<-sqrt(2*pi)*(betasq/2)^(-k-0.5)
PPBF<-(q*num)/(Q*denom)
return(PPBF)},
#Exponential prior form
EPBF={
c=hyper
#normalising constant for prior
r=c/(2*(exp(-c*a/2)-exp(-c*b/2)))
#integrand
library("GeneralizedHyperbolic")
int<-rep(NA,nSNP)
for(i in 1:nSNP){
if(abs(betas[i])>=0.01){
intA<-pgig(a+vars[i], param = c(betasq[i],c,1/2))
intB<-pgig(b+vars[i], param = c(betasq[i],c,1/2))
int[i]<-intB-intA }}
#BF
num<-2*exp(c*vars/2)*besselK(sqrt(c*betasq), 0.5, expon.scaled = FALSE)
denom<-sqrt(2*pi)*(c/betasq)^0.25
EPBF<-(r*num*int)/(Q*denom)
return(EPBF)},
#Hybrid prior form
HPBF={
d=hyper[1]
k=hyper[2]
```

```
#normalising constant for prior
inc.gamma.part<-gamma(-k-1)*(pgamma(d/(2*(vars+a)), -k-1)-pgamma(d/(2*(vars+
b)), -k-1))
s<-(d/2)^ (-k-1)/inc.gamma.part
#BF
num<-gamma(-k-0.5)*(pgamma((betasq+d)/(2*(vars+a)), -k-0.5)-pgamma((betasq+d)
/(2*(vars+b)), -k-0.5))
denom<-sqrt(2*pi)*((betasq+d)/2)^ (-k-0.5)
HPBF<-(s*num)/(Q*denom)
return(HPBF)},
#Reciprocal prior form
RPBF={
#approximation of sum for incomplete gamma with 0 term:
abfun<-function(n){
((-1)^ n)*(((b+vars)/2)^ n-((a+vars)/2)^ n)/(n*factorial(n)) }
absum<-0
for(i in 1:1000){
absum<-absum+abfun(i)}
#normalising constant for prior
t<-1/(log((b+vars)/(a+vars))+absum)
#integrand
ap<-pnorm(sqrt(a+vars)-abs(betas)/sqrt(a+vars))
am<-pnorm(-sqrt(a+vars)-abs(betas)/sqrt(a+vars))
bp<-pnorm(sqrt(b+vars)-abs(betas)/sqrt(b+vars))
bm<-pnorm(-sqrt(b+vars)-abs(betas)/sqrt(b+vars))
int<-bp-ap+(bm-am)*exp(2*abs(betas))
#BF
RPBF<-(t*exp(-abs(betas))*int)/(Q*abs(betas))
return(RPBF)},
)}
```
